# Design of a Randomized Placebo-Controlled Trial to Evaluate the Anti-inflammatory and Senolytic Effects of Quercetin in Patients Undergoing Coronary Artery Bypass Graft Surgery

**DOI:** 10.3389/fcvm.2021.741542

**Published:** 2021-10-20

**Authors:** Olina Dagher, Pauline Mury, Pierre-Emmanuel Noly, Annik Fortier, Guillaume Lettre, Eric Thorin, Michel Carrier

**Affiliations:** ^1^Department of Surgery, Faculty of Medicine, Université de Montréal, Montreal, QC, Canada; ^2^Research Centre, Montreal Heart Institute, Université de Montréal, Montreal, QC, Canada; ^3^Department of Cardiac Sciences, Cumming School of Medicine, University of Calgary, Calgary, AB, Canada; ^4^Montreal Health Innovations Coordinating Center, Université de Montréal, Montreal, QC, Canada; ^5^Department of Medicine, Faculty of Medicine, Université de Montréal, Montreal, QC, Canada

**Keywords:** coronary artery bypass grafting, quercetin, inflammation, senescence, senolytic, angiopoietin-like 2, randomized controlled trial

## Abstract

**Background:** Following an acute coronary syndrome, patients display an elevated inflammatory profile, promoted in part by cellular senescence. For patients requiring a coronary artery bypass (CABG) surgery, exposure to the surgical intervention and cardiopulmonary bypass further exacerbate their residual inflammation. Experimental evidence identified quercetin, a natural senolytic drug, as a cardioprotective agent against inflammatory injuries. The Q-CABG study aims to explore the efficacy of quercetin to reduce inflammation, myocardial injury and senescence in patients undergoing CABG following an acute coronary syndrome.

**Methods:** Q-CABG is a phase II, prospectively registered, randomized, double-blind and placebo-controlled clinical trial. Recruited patients awaiting CABG surgery at the Montreal Heart Institute (n = 100) will be randomly assigned in a 1:1 ratio to receive either quercetin supplementation (500 mg twice daily) or placebo, starting 2 days before surgery and until the seventh postoperative day. The primary endpoint examines the effects of quercetin on blood inflammatory cytokines and markers of myocardial injury and senescence in this patient population. Blood samples will be taken at four time points: baseline, postoperative day 1, postoperative day 4 and at hospital discharge, or after a maximum of seven postoperative days. The secondary endpoint is the assessment of endothelial (dys) function by looking at *ex vivo* vascular reactivity and mRNA expression of endothelial cells from the wall of discarded segments of internal mammary artery.

**Discussion:** The preventive intake of quercetin supplementation may help limit the vigorous inflammatory response triggered by CABG and subsequent postoperative complications in patients suffering from an acute coronary syndrome. In an exploratory way, quercetin supplementation could also improve endothelial function by eliminating senescent vascular endothelial cells. The results of this trial should provide valuable information regarding a novel approach to improve biological, and potentially clinical, outcomes post CABG.

**Clinical Trial Registration:** ClinicalTrials.gov, Identifier NCT04907253.

## Introduction

Many patients on potent agents addressing modifiable cardiovascular risk factors, such as hypertension, hyperlipidemia or diabetes, still suffer from cardiovascular complications, including acute coronary syndromes (ACS). It indicates that other pathological mechanisms responsible for this residual risk are not yet targeted by current medical regimens. Inflammation has emerged as a central mediator of endothelial dysfunction, and by extension, vascular disease (1). High levels of inflammatory markers, such as C-reactive protein, were associated with left ventricular dysfunction (2), higher cardiovascular event rates (3) and worse clinical prognosis (4) following an acute myocardial infarction, independent of traditional risk factors. These observations opened the door to testing anti-inflammatory agents as a new therapeutic adjunct in the management of cardiovascular disease (5). Recently, three large-scale randomized controlled trials, CANTOS (6), CIRT (7), and COLCOT (8), explored these benefits in the context of an ACS. While the magnitude of the observed outcomes differed between these trials, altogether, their findings suggest that a conventional management coupled with inflammation inhibition could maximize vascular benefit and clinical outcomes (6–8).

One common exclusion criterion in these trials was the need to undergo coronary artery bypass grafting (CABG) as a revascularization strategy. However, this patient population is exposed to a further complex inflammatory response from the additive stresses of the acute myocardial ischemia, the reperfusion and the extracorporeal circulation. It is increasingly recognized that the vigorous inflammatory response triggered by on-pump CABG has important clinical implications as it may contribute to the genesis of common postoperative complications. These can range from vasoplegia, coagulopathy, cardiac arrhythmias, and acute kidney injury to ongoing organ hypoperfusion and multi-organ dysfunction syndrome (9). It may even exert adverse vascular effects on native or grafted coronary vessels, which may impair revascularization (10). Factors influencing the severity of the clinical sequelae of this inflammatory response, and, in particular, the reasons why certain patients develop life-threatening perioperative complications, are currently not well-understood. Intuitive contributing events include patients' frailty, their co-morbidities, the presence of a preoperative infection or cardiogenic shock, as well as the length of pump time, hypothermia, and the amount of blood loss or transfusion. Combined together, these triggers form a “multiple-hit” scenario. Therefore, the contribution of this inflammatory response to patients' outcomes deserves attention.

In parallel to inflammation, an age-related deterioration in cardiovascular structure and function naturally occurs with time. This biological aging, termed senescence, leads to increased susceptibility to injurious stimuli, such as metabolic stress and ischemic or reperfusion injuries (11). Our team previously studied the perioperative evolution of the inflammatory and senescent profiles in 47 patients undergoing cardiac surgery (12). It showed that plasma angiopoietin-like 2 (ANGPTL2) levels, which vascular wall expression correlates with that of established markers of inflammation and senescence in these patients (13), remained at above-baseline levels in older hypertensive patients, unlike the high-sensitivity C-reactive protein (hs-CRP) levels which decreased, on average, in all patients. This study suggests that corrective cardiac surgery alone does not alleviate the senescence status in higher risk patients.

The potential predictive value of a decrease in senescence burden on postoperative outcomes has not yet been explored, but there is clear evidence that senescent cells in the heart contribute to functional decline including decreased contractility and impaired diastolic function, as well as left ventricular hypertrophy (14–17), while in arteries, it is associated with endothelial dysfunction and atherogenesis (13, 18, 19). As the elderly population continues to grow, the need for interventions to combat this age-related cardiovascular decline is becoming increasingly pressing. A novel and exciting approach to intervene is by using senolytics, small molecules capable of eradicating senescent cells (20). One of the first senolytic drugs to be identified was quercetin, a natural flavonoid ubiquitously present in fruits, vegetables, nuts, tea, and red wine (21). Traditionally, quercetin was mainly known for its anticancer potential and apoptosis-inducing effects *via* Bcl-2 and Bax regulation (22), but was later found to exert many other effects, including anti-inflammatory, anti-oxidant, anti-diabetic, anti-histaminic, anti-bacterial, and neuroprotective (23). In addition, extensive data from experimental and clinical studies showed that quercetin elicits a wide range of cardioprotective biological activities (24). Clinical trials looking at the therapeutic potential of this multifaceted molecule in cardiovascular diseases are, however, lacking (24). An appreciable advantage of quercetin compared to other senolytic agents, such as dasatinib, is the absence of clinically significant side effects, making it a safe molecule to study in humans (25).

The current phase II randomized placebo-controlled trial explores the efficacy of quercetin in 100 patients undergoing CABG following an ACS. The primary objective of this study is to evaluate the effects of quercetin on the resolution of systemic inflammatory response and myocardial injury as well as the burden of senescence in this patient population. The secondary objective is to determine the effects of quercetin on endothelial function *ex vivo* by looking at vascular reactivity and mRNA expression of endothelial cells from the wall of discarded segments of the internal mammary artery (IMA). We hypothesize that patients exposed to quercetin perioperatively will demonstrate a lower profile of inflammation, senescence and myocardial injury at hospital discharge, and as a result, will have an improved endothelial function.

## Methods and Analysis

### Study Design

Q-CABG (ClinicalTrials.gov, NCT04907253) is a phase II, single center, prospective, randomized, double-blind, allocation-concealed and placebo-controlled study evaluating the anti-inflammatory and senolytic effects of a short-course (hospital stay) treatment of quercetin (500 mg P.O. bid) vs. placebo in patients undergoing CABG. The trial protocol was approved by the institutional review board and by Health Canada. Q-CABG will be conducted at the Montreal Heart Institute (MHI) in Montreal, Quebec, Canada. The MHI is a regional referral center dedicated for cardiovascular care, with a catchment area of ~ four million people. Close to 2,000 heart surgeries are performed every year.

**Figure 1:**
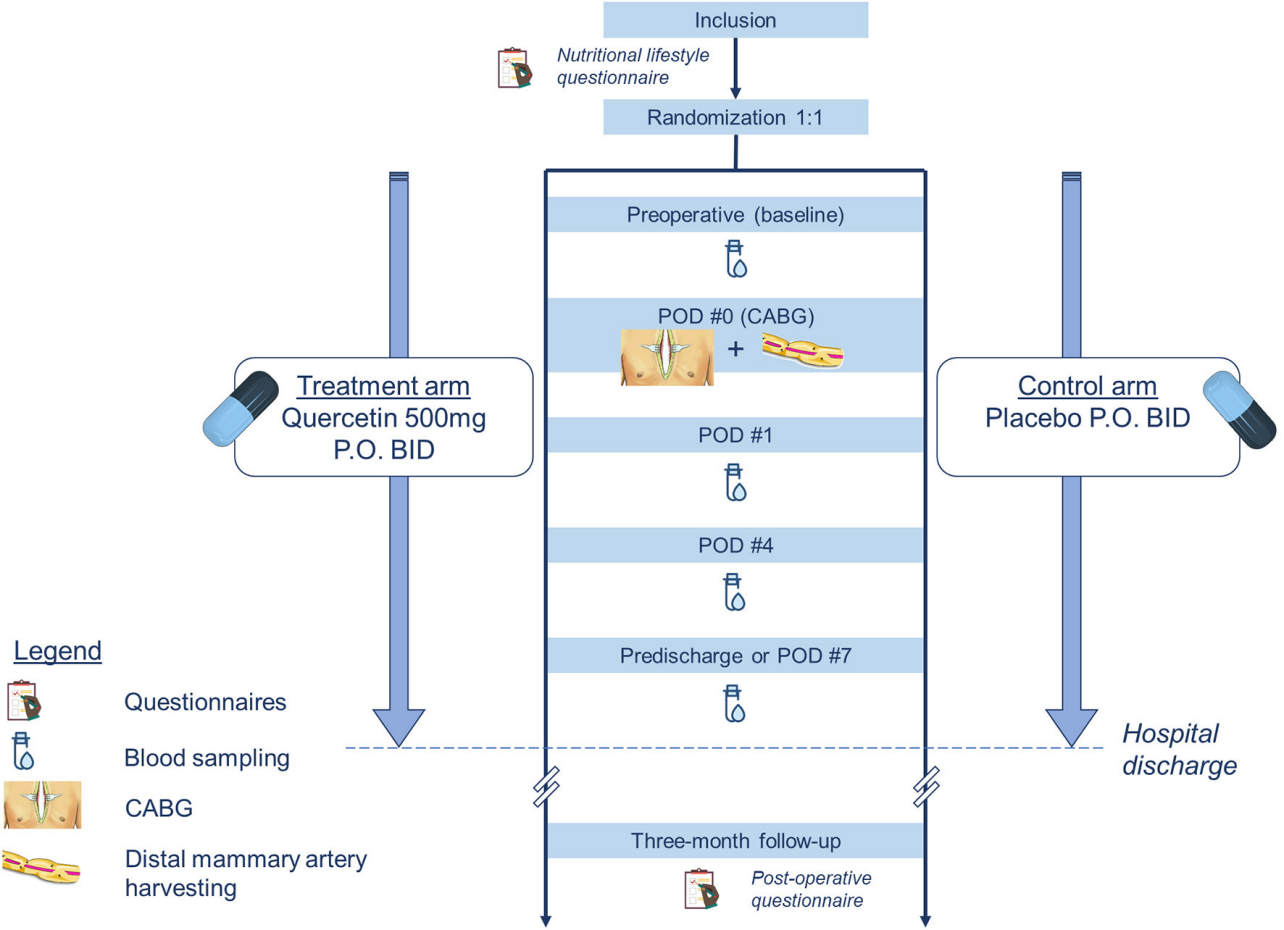
Flow-chart of the study design. CABG, coronary artery bypass grafting; PO, *per os*; POD, post-operative day.

This clinical trial is designed to recruit 100 adults scheduled to undergo an inpatient CABG at the MHI following an ACS (Figure 1). The definition of an ACS includes the diagnosis of unstable angina, non-ST elevation myocardial infarction (NSTEMI) or ST elevation myocardial infarction (STEMI). Participants will be recruited at the coronary care unit or the step down unit of the MHI. Patients are considered for inclusion if they meet the criteria as defined below. The patient allocation ratio is 1:1. Participants will be treated according to national guidelines (26) that include optimal medical therapy as determined by their primary care team.

### Study Population

Inclusion criteria target 18 years old and older patients with a diagnosis of ACS in the last month. Key exclusion criteria include a combined heart surgery, documented severe kidney or liver failures, immunosuppressed patients or patients with a systemic auto-immune disorder, as well as pregnant or lactating patients. Given that surgical strategy is often unknown at the time of recruitment, off-pump cases are not excluded. However, this parameter will be taken into account since these patients are expected to have a different inflammatory profile compared to that of patients exposed to cardiopulmonary bypass (27). Co-administration of vitamin K antagonists is not considered an exclusion criterion because clinically relevant interactions with quercetin remain anecdotal (28). Nevertheless, international normalized ratio values will be closely monitored in patients who receive a vitamin K antagonist. Pain management is left at the discretion of the primary care team. Administration of non-steroidal anti-inflammatory drugs is not an exclusion criterion since they are part of the routine pain treatment plan. Complete inclusion and exclusion criteria are outlined in Table 1.

**Table 1:** Q-CABG inclusion and exclusion criteria.

**Inclusion criteria**	**Exclusion criteria**
Males and females of at least 18 years of age capable and willing to provide informed consent in either English or French without the need of an interpreter	Patient currently taking a natural supplement containing quercetin or its metabolites
Patient must have suffered a documented ACS* within the last 30 days	Patient with a history of an allergic reaction or significant sensitivity to quercetin, another flavonoid, niacin or ascorbic acid
Patient must be treated according to national guidelines (including anti-platelet therapy, statin, renin-angiotensin-aldosterone system inhibitor, beta-blocker and planned revascularization procedure when indicated)	Patient is currently using or plans to begin chronic systemic steroid therapy (oral or intravenous) during the study (topical or inhaled steroids are allowed)
Patient must be scheduled to undergo an inpatient (urgent or semi-urgent) isolated CABG at the Montreal Heart Institute	Patient is immunosuppressed or was diagnosed with a systemic auto-immune disorder
Patient must be able and willing to comply with the requirements of this study protocol	Patient currently in cardiogenic shock or with hemodynamic instability
	Patient requiring an emergency CABG
	Patient undergoing a CABG combined with another cardiac intervention (such as a valvular repair or replacement)
	Patient with a history of breast cancer or other estrogen-dependent cancer
	Patient with a history of cirrhosis, chronic active hepatitis or severe hepatic disease
	Patient with any of the following as measured within the past 30 days, and determined to be non-transient through repeat testing: ALT or AST > 2 times the ULN, an eGFR <30 mL/min.
	Patient requiring to take a fluoroquinolone post-operatively
	Female patient who is pregnant, or breastfeeding or is considering becoming pregnant during the study or for 6 months after the last dose of study medication
	Patient is considered by the investigator, for any reason, to be an unsuitable candidate for the study

ACS, acute coronary syndrome; ALT, alanine aminotransferase; AST, aspartate aminotransferase; CABG, coronary artery bypass grafting; eGFR, estimated glomerular filtration rate; ULN, upper limit of normal. *, The definition of an ACS includes the diagnosis of unstable angina, non-ST elevation myocardial infarction (NSTEMI) or ST elevation myocardial infarction (STEMI).

### Recruitment

Patients aged 18 years or older admitted to the MHI for an ACS diagnosed within the past 30 days and expected to undergo an inpatient CABG are eligible to participate. The indication for CABG will be determined by the primary care team in the presence of angiographically documented multi-vessel coronary artery disease not amenable to coronary angioplasty and/or better served by surgery as *per* the national Canadian guidelines on revascularization (26). After the patient is screened for eligibility, she/he will receive initial study information and will be provided with a copy of the consent. After a period of reflection, patients are invited to meet again with the research physician to discuss any remaining questions and sign the informed consent if interested. If they choose to participate, they will fill a lifestyle habits and sedentary behavior questionnaire (Table 2). This questionnaire was obtained by combining elements from three validated tools: the Standardized Mini-Mental State Examination (SMMSE), the Sedentary Behavior Questionnaire (SBQ) and the lifestyle questionnaire derived from the Educoeur study (29–31). It will provide us with general information on the diet and exercise habits of the study participants before their hospital admission. The questions extracted from the SMMSE are used to confirm patients' cognitive orientation before surgery.

**Table 2:** Lifestyle habits and sedentary behavior questionnaire.

**1. Cognitive orientation**										
(a) What year is this?										
(b) What season is this?										
(c) What month is this?										
(d) What is today's date?										
(e) What day of the week is this?										
(f) What country are we in?										
(g) What province are we in?										
(h) What city/town are we in?										
(i) What is the name of this building?										
(j) What room are we in?										
**2. Smoking habits**										
(a) I smoke everyday.			How many cigarettes/day:			Started at age:				
(b) I don't smoke every day.			How many cigarettes/week:			Started at age:				
(c) I don't smoke but I've smoked every day in the past.			How many cigarettes/day:			Started at age:			Stopped at age:	
(d) I never smoked.										
**3. Sedentary behavior**	**Time spent on a typical weekday**									
	**None**	**-15min**	**15–30 min**	**30–60 min**	**1–2 h**	**2–3 h**	**3–4 h**	**4–5 h**	**5–6 h**	**>6 h**
1. Sitting at work										
**During your leisure time**										
2. Sitting watching television										
3. Sitting on the computer										
4. Sitting during meals										
5. Sitting listening music or reading a book										
6. Sitting with friends or family										
7. Sitting and driving a car, bus or train										
8. Sitting during leisure time										
9. Taking a nap										
**4. Nutritional habits**	**1**	**2**	**3**
1. How many meals do you eat per day?			3 meals/day			A fourth			1-2 meals/day	
2. Do you eat between meals?			No, I have a snack when I need it			I eat in the evening			I often nibble between meals	
3. Do you eat prepared foods?			<1/week			1–2/week			≥3/week	
4. How many portions of vegetables do you eat?			≥4/day			2–3/day			≤ 1/day	
5. How many portions of fruits do you eat?			≥3/day			2/day			≤ 1/day	
6. How often do you eat deli or fatty meats?			<1/week			1–2/week			≥3/week	
7. How often do you eat baked products (muffins, brioche, croissant, etc.)?			≤ 1/week			2–3/week			≥4/week	
8. How often do you eat sweets?			≤ 3/week			4–5/week			everyday	
9. Do you drink sweet drinks, including 0% calories?			250mL or less/day			250mL/day			250mL or more/day	
			1			2			3	
10. What is your average alcohol consumption?			<1/day			2/day			>2/day	

### Assignment of Interventions (Randomization and Blinding)

Enrolled participants are randomly assigned to (i) the intervention arm, in which they receive quercetin supplementation or to (ii) the control arm, in which they receive a placebo capsule, both given twice daily. The randomization list is computer-generated by an unblinded biostatistician from the Montreal Health Innovations Coordinating Center (MHICC). The allocation sequence uses a 1:1 ratio and a blocking schema with blocks of size two, four, and six. During the conduct of the study, patients, investigators and health care staff are blinded to the individual treatment assignments. All doses of both quercetin and placebo are dispensed in opaque bottles for nursing inpatient administration. The capsule covers are green for both treatment arms in order to ensure proper concealment of group allocation.

### Medication

The trial medication and matching placebo were provided free of charge by Advanced Orthomolecular Research (AOR) Inc. (Calgary, Alberta, Canada), which had no role in the design or conduct of the trial. The composition of the quercetin capsules is as follows: 92% quercetin, 5% Arbocel A300 (microcrystalline cellulose), and 3% sodium stearyl fumarate. The quercetin is extracted from the *Dimorphandra mollis* flowers in a water solution then purified with ethanol precipitation. The resultant dehydrated powder has a pH and pKa of 7.4 and 7.17, respectively, in aqueous solution. For the placebo, quercetin is simply replaced by Arbocel A300 (97%). The capsule covers are made of pharmaceutical grade cellulose. All capsules (quercetin and placebo) are stocked at the hospital pharmacy in opaque pill boxes.

In terms of pharmacokinetics, quercetin undergoes first-pass metabolism by the liver where it is almost completely metabolized by glucuronidation, methylation, or sulfonylation (24). Quercetin's metabolism seems to be dependent on apolipoprotein E phenotype (32, 33) but peak plasma concentration is reached within 4 h in most patients (24).

Safety of patients will be assessed on a continuous basis. Quercetin is a well-tolerated substance. It is approved by the U.S. Food and Drug Administration, Health Canada and the European Medicines Agency, among many other public health agencies, and sold as an over-the-counter natural product in Canada. Mild secondary effects have been reported anecdotally (25). Trial medication will be stopped if patients develop an allergic reaction or an undesirable effect. The latter will be reported in the chart by the research team and communicated to the primary care team. It will also be stopped if the patient needs to take a fluoroquinolones. Quercetin was reported to bind to the DNA gyrase enzyme in bacteria, which could competitively inhibit that class of antibiotics (34).

### Intervention

All participants will be managed by their own primary care team. They will receive conventional optimal medical therapy and will undergo CABG as *per* national guidelines. The 50 patients randomized to the treatment arm will receive capsules of quercetin at a dose of 500 mg P.O. bid (morning and evening doses). The other half participants allocated to the control group will receive a placebo, also twice daily, in the form of a powder capsule visually indiscernible from the trial medication. Treatment administration starts 2–3 days before planned surgery so that patients receive four–six doses pre-operatively. It will be continued after surgery until hospital discharge or a maximum of seven postoperative days (POD). Hospital stay will not be prolonged for the sole purpose of the study.

### Premature Discontinuation of Study Drug

Participants who discontinue treatment based on toxicity rules defined above, but who agree to stay in the study, will continue to be followed and additional data on secondary endpoints will continue to be collected according to the study flow chart. The same applies to patients who violate the research protocol for other reasons, such as missing one or more doses. Our aim is to preserve the integrity of randomization as much as possible throughout the entire study and in analysis. Therefore, an intention-to-treat principle will be applied.

### Post-treatment Phase

For both groups, the study treatment (quercetin or placebo) is stopped at hospital discharge. Pill boxes are returned to the hospital pharmacy for pill counting. Participants will be contacted or seen at their routine outpatient follow-up by a member of the research team, 6–12 weeks after surgery. They will be questioned about their post-operative recovery, their symptoms or complications (wound infection, recurrent angina, recurrent hospitalization, atrial fibrillation, stroke, etc.).

### Data Collection

Data will be derived from questionnaires, paper charts and electronic paper records and compiled in a spreadsheet. All data acquired during the study will be anonymized and documents related to data collection or analysis will be saved in a study folder. Only the study team has access to this specific study folder. The lifestyle questionnaire collects demographic data, including living situation, employment status, education and socio-professional level, as well as information about sedentary behavior, smoking and nutritional habits (Table 2). Clinical data includes general characteristics (age, sex, weight, height), comorbidities, home medications, in-hospital medications, hematological and biochemistry results, hemodynamic data (arterial pressure, heart rate, atrial fibrillation), surgical data (pump time, cross-clamp time), as well as echocardiographic parameters, if available. A complete data collection schedule is provided in Table 3.

**Table 3:** Data collection schedule.

**Events**	**Recruitment**	**First day of treatment (2–3 days before surgery)**	**Day of surgery**	**POD #1**	**POD #2**	**POD #3**	**POD #4**	**POD #5–6**	**Hospital discharge or POD #7 at the latest**
Consent	X								
Lifestyle habits and sedentary behavior questionnaire	X								
Randomization	X								
Study drug administration		X	X	X	X	X	X	X	X
Blood tests		X		X			X		X
Internal mammary artery sampling			X						

*POD, post-operative day*.

## Experimental Methods and Study Objectives

### Plasmatic Inflammatory Response – Primary Endpoint

The primary outcome measure is the temporal evolution of the inflammatory response in the first place, as well as the burden of myocardial injury and senescence. This will be evaluated with the variation in circulating blood levels of, firstly, hs-CRP as the most important parameter, and secondly myocardial high-sensitivity troponin T (hs-cTnT) and ANGPTL2, respectively.

Participants will have blood tests done at four time points in the perioperative period: at baseline (prior to administration of the study drug), 24h after surgery, 4 days after surgery and prior to hospital discharge or a maximum of 7 days after surgery (Figure 1). These intervals were chosen based on data previously published by Noly et al. (12). In this study, these inflammatory markers had a strong increase at 24h and peaked at 4 days in this patient population. Venous blood samples will be drawn peripherally and collected by the nursing staff on the ward. Time for blood collection is not fixed and is left at the discretion of the ward. Hs-CRP and hs-cTnT will be analyzed by the hospital laboratory together with the rest of the patients' biochemistry panels, if applicable. A separate specimen tube will be collected for the research laboratory to measure the serum ANGPTL2 using an ELISA kit (Cloud-Clone Corp., USA).

### Endothelial Function Assessment – Secondary Endpoint

The secondary outcomes relate to the endothelium of the IMA. Whenever possible, an unused discarded distal segment of the internal mammary artery harvested as a graft will be provided by the surgeon in the operative room. The harvesting technique of the graft, skeletonized or non-skeletonized, will be left at the discretion of the surgeon but will be taken into account at the time of results analysis. At the MHI, monopolar electrocautery is used as the primary instrument for IMA dissection, typically at a coagulation setting of 20 Electrosurgical Units (ESUs). The tissue sample is collected in a plastic tube containing sterile cell culture medium and placed on ice for immediate processing. The same segment of IMA will serve two purposes. First, we will evaluate endothelial function *ex vivo* by measuring vascular reactivity to acetylcholine (ACh). Second, we will quantify the burden of senescent cells in the vascular wall through the expression of mRNA.

### Vascular Reactivity

Regarding the evaluation *ex vivo* of the endothelial function, we will mount a freshly collected 2-mm segment of the IMA in a wire myograph filled with 10 mL of physiological salt solution (PSS), as previously described (35). Arteries will be pre-constricted with 10–30nM of U46619, a mimetic of the Endoperoxide prostaglandin PGH_2_ and acting as a thromboxane A_2_ receptor agonist. At the plateau of the constriction, we will add cumulative doses of ACh (10 doses from 1nM to 30mM) in order to assess the endothelium-dependent vasorelaxation. At the end of the experiment, we will maximally constrict the segment with 127mM KCl-PSS, in order to calculate the percent of constriction induced by U46619. This experiment will permit to obtain two indexes of endothelial (dys) function: *ACh-EC*_50_, the potency, which represents the concentration of ACh needed to reach 50% of the maximal relaxation, and, *E*_*max*_, the efficacy, which represents the maximal relaxation obtained at a plateau.

### Transcriptomic Signature

Secondly, we will evaluate the transcriptomic signature of the IMA. For this purpose, a special attention is needed to obtain good RNA quality: the tissue needs to be snap frozen as soon as possible after its excision to avoid losing RNA integrity. In contrast, the timing to retrieve the sample had no influence on vascular contraction and endothelium-dependent relaxation.

Firstly, for 12 samples randomly allocated by the pharmacist (six patients treated with quercetin vs. six patients taking placebo), we will run a single-nuclei RNA sequencing. All samples will be stocked at −80 degrees and processed at the same time (once recruitment of all patients is completed). Arteries will be gently crushed in a mortar into liquid nitrogen. In order to obtain single-nuclei, cells will be lysed on ice using a modified protocol from 10X Genomics (10X-Multiome Seq Protocol). Then, nuclei will be counted, loaded onto a 10X chip and processed on the 10X Chromium Controller for 3' Gene Expression experiment. Libraries will be prepared following manufacturer's instructions, and sequenced on NovaSeq S3 or S4 (Illumina) at Genome Quebec. Computational analysis will be done using Seurat toolkit (36) on R. Our focus will be the comparison of differentially expressed genes (DEGs) between treatment groups (quercetin vs. placebo) mainly on endothelial cells, but all others cell types will be considered in an exploratory way.

Secondly, for 12 different samples randomly allocated by the pharmacist (six patients treated with quercetin vs. six patients taking placebo), we will run a bulk RNA sequencing. All samples will be stocked at −80 degrees and processed in the same time (once recruitment of all patients is completed). Arteries will be gently crushed in a mortar into liquid nitrogen. The mRNA will be extracted from the powder using a standard protocol from Qiagen (RNeasy Mini Kit, Qiagen). Then, mRNA quantity and quality will be evaluated using the Bioanalyzer System (RNA 6,000 Nano Kit, Agilent) to assess the RNA Integrity Number. Library preparation and sequencing will be processed at the McGill Genome Centre. Computational analysis will be done using R. Our focus will be the comparison of pathway analysis between treatment groups (quercetin vs. placebo) using some databases such as Gene Ontology (GO) and/or Kyoto Encyclopedia of Genes and Genomes (KEGG).

## Statistical Analysis

### Sample Size

The primary endpoint for this study is the blood level change of hs-CRP over time, more specifically between the baseline value and 4 days after CABG. It is expected that a log transformation will be applied to the data before performing the analyzes. The sample size calculation was therefore performed with this assumption. Given the transformation of the data by the logarithm, the change between the two values, as well as the “difference” between the groups are expressed as a ratio:

(hs-CRP at postoperative *day* #4 (quercetin)/hs-CRP at baseline (quercetin)) ÷

(hsCRP at postoperative day #4 (placebo)/hs-CRP at baseline (placebo))

A 50% reduction in the change in hs-CRP would represent a significant clinical gain. Such a reduction, once transformed on the logarithmic scale, corresponds to a difference to be detected of 0.693 ((log (hs-CRP at postoperative day #4 (quercetin)) – log (hs-CRP at baseline (quercetin))) – ((log (hs-CRP at postoperative day #4 (placebo)) – log (hs-CRP at baseline (placebo))) = log (0.50) = – 0.693). Based on the data observed in a previous study (12), the standard deviation assumed for the ratio (hs-CRP at postoperative day #4/hs-CRP at baseline) log-transformed (*i.e*., the standard deviation for log (hs-CRP at postoperative day #4) -log (hs-CRP at baseline)), common to both groups, is 1.215 (1). A sample of 50 subjects *per* group (100 subjects in total) will detect a relative reduction of 50% in the change in hs-CRP in the quercetin group compared to the placebo group, with a two-tailed significance level of 0.05 and a power of 80%.

### Statistical Methods

Analyses will be performed on the intention-to-treat (ITT) population including all randomized participants analyzed according to the randomization scheme. A log transformation will be applied to the hs-CRP values prior to analysis. The changes in blood level values of hs-CRP, hs-cTnT and ANGPTL2 from baseline (before the first oral intake of the study drug) will be compared between the groups using repeated measures analysis of covariance (ANCOVA) including a term for the intervention group (quercetin or placebo), a term for time, a term for the interaction between the group and time, as well as an adjustment term for the baseline value. Comparison of hs-CRP levels at postoperative day #4 between the 2 groups (quercetin vs. placebo) will be the main point of comparison for the primary objective since it was used for sample size calculation.

The measurement of the relaxing endothelial function induced by ACh *ex vivo* (*i.e*., EC_50_ and E_max_ values) will be compared between groups using Student's tests. Regarding the single-nuclei and bulk RNA sequencing, differentially expressed genes (DEG) will be determined using the Seurat R toolkit (36). The *p* < 0.05 and |log2(FC)| >1 will be considered significant. Moreover, pathway analysis will be ran using Gene Ontology (GO) and Kyoto Encyclopedia of Genes and Genomes (KEGG) databases. False discovery rate (FDR) <0.05 and *p* < 0.05 will be considered significant.

Statistical analyses will be performed using SAS^®^ software (version 9.4 or later, Cary, NC).

### Data Monitoring

Data output will be stored in the statistical software for analysis. Informed consents and end-of-trial dates will be recorded in the patient's chart and signed paper forms will be stored within the hospital in a locked room. To be able to reproduce study results and to help future users understand and reuse data, all changes made to the raw data and all steps taken in the analysis will be documented. Source data will remain available in the electronic patient record. All research data, including patient material, will be archived for 25 years after the study has ended according to the guidelines for Advanced Therapy Medicinal Products (ATMPs). Research data will be stored using a study identification code for each participant. The key to the identification code list will only be available to the research team during the study and will be documented and safeguarded by the principal investigator according to research guidelines after completion of the study. No patient identification details will be reported in publications.

## Discussion

Despite the advent of revascularization strategies, ACSs are still a major cause of mortality and morbidity worldwide (37). Furthermore, recurrent ischemic cardiovascular events remain a crucial problem to solve. Secondary prevention strategies usually combine physical rehabilitation with an optimal pharmacological regimen for reduction of traditional cardiovascular risk factors. The findings from the COLCOT trial showed that there is even more room to act by tackling systemic inflammation. We want to push the paradigm further by testing an agent with both anti-inflammatory and anti-aging properties. Aging contributes to the deterioration of cardiovascular function through the accumulation of senescent cells, a phenomenon that is further amplified in patients with structurally and functionally “vulnerable” hearts (11). Introducing a safe and well-tolerated senolytic agent as a preventive strategy could be of significant relevance in these patients.

As discussed in recent reviews and meta-analyses, quercetin exerts many cardioprotective effects (24, 38–41). In both healthy and metabolically compromised human subjects, quercetin supplementation significantly reduced direct or indirect markers of cardiovascular disorder such as glycemia, total cholesterol, LDL-cholesterol, blood pressure and CRP levels (24, 38–41). Despite these promising effects of quercetin, clinical trials are still lacking in this field (24). Q-CABG is designed to investigate the efficacy of quercetin in patients undergoing CABG. “Efficacy” will be evaluated as the capacity of quercetin to reduce the burden of systemic inflammation, myocardial injury and senescence, as well as improve endothelial function of the IMA. Our study brings many aspects of novelty. To the best of our knowledge, Q-CABG is the first randomized controlled trial to propose quercetin as an adjunctive therapy for patients undergoing CABG. In addition, the study design is unique in that it will simultaneously gather preliminary data on biological, functional and clinical outcomes. It will also provide a critical understanding of the endothelial (dys) function of the most important conduit in coronary surgery, namely the IMA, and the impacts of the inflammasome activation on its function; our laboratory previously showed that the endothelium of the IMA mirrors patient's disease history and is subjected to premature senescence in relation to the burden of disease (42). Furthermore, this project could help us draw a more complete characterization of the senescence associated secretory phenotype profile in response to cardiopulmonary bypass at both clinical and cellular levels. This represents a largely unexplored topic.

This trial is not without challenges and limitations. First, one could argue that, unlike hs-CRP, and ANGPTL-2 which are inflammatory markers, quercetin is not an inflammation-specific agent. A better way to describe the pharmacological activity of quercetin would be to say that it is a multifaceted agent. However, quercetin does have well documented anti-inflammatory effects in patients with chronic inflammatory diseases (43, 44). For example, it was shown to decrease the expression of the IκBα gene in blood mononuclear cells of patients with coronary artery disease (45). This is especially relevant as *I*κ*B*α is involved in the activation of NF-κB, which, in turn, controls the transcriptional regulation of ANGPTL-2 (46). In addition, biomarker selection for our primary outcome was mainly driven by a prior study done at our center which documented the perioperative evolution of hs-CRP and ANGPTL-2 in a cohort of 47 cardiac surgery patients (12). We used the data observed by Noly et al., to calculate our sample size, assuming a 50% relative reduction in hs-CRP levels would represent a significant clinical gain. Hs-CRP is a routinely used marker and has a prognostic role in ACS (47). Therefore, we thought it would be of relevance in our protocol. Another limitation of this study is the relatively low absorption and bioavailability of quercetin (48). Comparative bioavailability studies have also reported substantial interindividual variations (48). This may explain the differences in the observed effects of quercetin among various clinical studies (24). In Canada, the recommended maximum daily dose of quercetin is 1,200 mg and intra-venous formulation is not approved by Health Canada. Therefore, since the optimal dosing strategy of quercetin remains unknown, we assumed that a dose of 1,000 mg daily would be a good balance between proven safety and accepted efficacy. In this era of Enhanced Recovery after Surgery (ERAS) protocols (49), it is not surprising to see patients going home as early as 3 days after CABG. Since participation in the trial will not delay patients' hospital stay, it is expected that the third time measure will not always be feasible on the fourth postoperative day. In these cases, the last blood test will be drawn as close as possible to hospital discharge. From a surgical perspective, the preferred harvesting technique for IMA is operator-dependent. At the MHI, both skeletonized and non-skeletonized approaches are used with the electrosurgical device typically set at 20 ESUs. Preliminary observations done at our laboratory showed that despite delicate handling of the IMA, tissue injuries caused by heat dissipation or direct burn from the electrosurgical blade tip can happen and compromise vascular wall integrity, as well as endothelial function. This might alter vascular reactivity to ACh. Another related challenge is maintaining IMA viability: our methodological validations have shown that while vascular reactivity is robust, mRNA integrity is very vulnerable to disintegration if not processed promptly enough.

## Conclusion

Q-CABG is a double-armed randomized clinical trial to study the efficacy of a natural flavonoid, quercetin, to decrease the inflammatory response, myocardial injury, and senescent burden after CABG in the context of an ACS. This study repurposes a safe and natural agent with proven cardiovascular benefits as an additional therapy to the armamentarium of ACS management. The results of this trial should provide valuable information regarding a novel approach to improve biological, and potentially clinical, outcomes post CABG.

## Trial Status

Recruitment started in June 2021. The current protocol is version 7 of 09-06-2021. Patient recruitment is estimated to be completed in April 2022.

## Ethics Statement

The studies involving human participants were reviewed and approved by Institutional Ethics Board, Montreal Heart Institute; and Natural and Non-prescription Health Products Directorate (NNHPD), Health Canada. The patients/participants will provide their written informed consent to participate in this study.

## Author Contributions

OD and PM: drafted the manuscript and designed the figures, with input from all authors. All authors participated in the design of the study and its main conceptual ideas, provided critical revisions to the article, and approved the final version for publication.

## Funding

This work was supported by the Canadian Institutes of Health Research [PJT 166110 and PJT 162446 to ET]; and the Foundation of the Montreal Heart Institute [ET]. PM is a post-doctoral scholar from the *Fonds de la recherche du Québec*.

## Conflict of Interest

The authors declare that the research was conducted in the absence of any commercial or financial relationships that could be construed as a potential conflict of interest. The prefilled capsules (quercetin and placebo) were provided by Advanced Orthomolecular Research (AOR) Inc. (3,900 12th Street Northeast, Calgary, Alberta, T2E 8H9, Canada) free of charge. However, the authors, nor their affiliated institutions, have received any form of financial compensation from AOR Inc.

## Publisher's Note

All claims expressed in this article are solely those of the authors and do not necessarily represent those of their affiliated organizations, or those of the publisher, the editors and the reviewers. Any product that may be evaluated in this article, or claim that may be made by its manufacturer, is not guaranteed or endorsed by the publisher.
